# Selenium-Ethylene Interplay in Postharvest Life of Cut Flowers

**DOI:** 10.3389/fpls.2020.584698

**Published:** 2020-12-17

**Authors:** Lucas C. Costa, Luana M. Luz, Vitor L. Nascimento, Fernanda F. Araujo, Mirelle N. S. Santos, Christiane de F. M. França, Tania P. Silva, Karen K. Fugate, Fernando L. Finger

**Affiliations:** ^1^Departamento de Fitotecnia, Universidade Federal de Viçosa, Viçosa, Brazil; ^2^Laboratório de Genética e Biotecnologia – Campus Capanema, Universidade Federal Rural da Amazônia, Capanema, Brazil; ^3^Setor de Fisiologia Vegetal – Departamento de Biologia, Universidade Federal de Lavras, Lavras, Brazil; ^4^Departamento de Tecnologia Agroindustrial e Socioeconomia Rural, Universidade Federal de São Carlos, Araras, Brazil; ^5^Instituto de Ciências Agrárias, Universidade Federal dos Vales do Jequitinhonha e Mucuri, Unaí, Brazil; ^6^USDA-ARS, Edward T. Schafer Agricultural Research Center, Fargo, ND, United States

**Keywords:** ethylene inhibitors, vase life, flower quality, preservative solutions, Se metabolism

## Abstract

Selenium (Se) is considered a beneficial element in higher plants when provided at low concentrations. Recently, studies have unveiled the interactions between Se and ethylene metabolism throughout plant growth and development. However, despite the evidence that Se may provide longer shelf life in ethylene-sensitive flowers, its primary action on ethylene biosynthesis and cause-effect responses are still understated. In the present review, we discuss the likely action of Se on ethylene biosynthesis and its consequence on postharvest physiology of cut flowers. By combining Se chemical properties with a dissection of ethylene metabolism, we further highlighted both the potential use of Se solutions and their downstream responses. We believe that this report will provide the foundation for the hypothesis that Se plays a key role in the postharvest longevity of ethylene-sensitive flowers.

## Introduction

Selenium (Se) is an essential nutrient for humans, bacteria, and most of the chlorophyte species (Lobanov et al., 2009; Nancharaiah and Lens, 2015). In higher plants, the beneficial effect of Se occurs in a concentration-dependent manner (Hawrylak-Nowak et al., 2014; Saidi et al., 2014; Boldrin et al., 2016; Sattar et al., 2019). At low concentrations, ranging from 0.1 to 1.0 mg L^–1^, Se stimulates plant growth and activates components of the reactive oxygen species (ROS) scavenge system, thereby protecting against multiple abiotic and biotic stresses (Feng et al., 2013; Ahmad et al., 2016; Lapaz et al., 2019). On the other hand, Se can be also toxic at concentrations ranging from 1 to 5 mg L^–1^, but the degree of tolerance varies among species (Freeman et al., 2010; Feng et al., 2013). Biological functions of Se occur primarily through selenoproteins which contain this element as part of the amino acids, selenocysteine (SeCys) and selenomethionine (SeMet) (Daniels, 1996), but also as a component of antioxidants, co-enzymes, specialized metabolites, and lipids (Khan M.I.R. et al., 2014; Khan N.A. et al., 2014). Therefore, the multiple presence of Se in plant metabolites evidences the unlimited possibilities of its action on plant metabolism, which has not been entirely explored.

Ethylene is a plant hormone mainly known for its role in affecting leaf and flower senescence, and fruit ripening. However, this simple gaseous molecule is also involved with other elemental processes throughout the plant’s life cycle, including seed germination (Corbineau et al., 2014; Miransari and Smith, 2014; Wilson et al., 2014), root initiation and development (Ivanchenko et al., 2008; Lima et al., 2009; Huang et al., 2013), floral development (O’Neill, 1997; Wuriyanghan et al., 2009), sexual determination (Iwahori et al., 1970; Yamasaki et al., 2001; Salman-Minkov et al., 2008), fruit ripening (Giovannoni, 2001; Barry and Giovannoni, 2007; Lim et al., 2007; De Martinis et al., 2015), plant senescence (Kim et al., 2014; De Martinis et al., 2015; Ueda and Kusaba, 2015), and response to biotic and abiotic stresses (Morgan and Drew, 1997; Wang et al., 2007; Lin et al., 2013; Steffens, 2014). Recently, several studies have uncovered evidence of a relationship between Se and ethylene metabolism in plants (Malorgio et al., 2009; Iqbal et al., 2015; Zhu et al., 2017; Hajiboland et al., 2019; Malheiros et al., 2019). In this vein, Malheiros et al. (2019) demonstrated that Se partially inhibits ethylene biosynthesis in roots of rice seedlings. Likewise, Iqbal et al. (2015) evidenced that Se inhibits 1-aminocyclopropane-1-carboxylate synthase (ACS) activity in wheat, the main limiting step of ethylene production in higher plants.

The production of flowers is one of the most important segments of horticulture in the field of agroindustry in many countries. The increased demand for high-quality products, however, requires postharvest technologies to improve floral vase life longevity. In recent years, the biological importance of ethylene on ornamental plant production and development of methods to alleviate its deleterious consequences in the overall ornamental value have been well explored. Nevertheless, many chemicals currently used to lessen ethylene responses present raised environmental and public health concerns. Thus, the development of methods that are environmentally friendly has become crucial (Scariot et al., 2014). Selenium presents suitable proprieties to be an eco-friendly (Cochran et al., 2018) and inexpensive (Haug et al., 2008) alternative to composing ethylene-sensitive flower preservative solutions. Recently, it was demonstrated that Se (6 mg L^–1^) increases the vase life of Easter Lily (*Lilium longiflorum*) by alleviating cell damage via the ROS scavenging system and osmotic adjustment (Lu et al., 2020). However, it seems that Se may have additional functions affecting the postharvest life of cut flowers that have yet to be investigated. Based on the current literature, herein we propose a novel model of interaction between Se metabolism and ethylene biosynthesis, which likely underlies positive consequences on postharvest life of cut flowers.

## An Overview of Se Chemical Characteristics and Metabolism

As part of the chalcogen group of chemical elements, Se is normally found in soils at concentrations from 0.01 to 2.0 mg kg^–1^ (Fordyce, 2005). This element exists in different oxidative states in soil conditions, such as elemental selenium (Se^0^), selenide (Se^2–^), thioselenate (Se_2_O_3_^2–^), selenite (SeO_3_^2–^), and selenate (SeO_4_^2^) (Neal et al., 1987; White et al., 2004). Among the different forms of Se, selenate is the most soluble and bioavailable for plants; additionally, it is the most predominant form of transport through the xylem, as compared to selenite (Asher et al., 1977; Gupta and Gupta, 2017). The essentiality of Se in plants has not been proven, but it seems to affect several aspects of plant metabolism. Discovered in 1817, this trace element is actively absorbed by root cells through the sulfur (S) transporters SULTR1;2 and SULTR1; however SULTR1;2 seems to be the preferential transporter for the uptake of Se (Gupta and Gupta, 2017). Once absorbed, all synthesized organoselenium compounds are derived from pathways associated with S metabolism (Terry et al., 2000) and accumulate in roots (Galeas et al., 2007), leaves, stems (Liang et al., 2019), flowers (Quinn et al., 2011), and seeds (Liang et al., 2019).

The metabolism of Se is partially dependent on chloroplast metabolic machinery, where the reduction of selenate to selenite occurs under the sequential action of two enzymes that incorporate Se into amino acids (Terry et al., 2000). The accumulation of selenoamino acids allows non-specific incorporation of SeCys or SeMet in plant proteins since SeCys insertion machinery has allegedly been lost through evolution (Lobanov et al., 2009; Pilon-Smits and Quinn, 2010). Moreover, selenoamino acids can be converted to volatile compounds or Se^0^ from the action of enzymes, such as methionine *S-*methyltransferase (Tagmount et al., 2002; Gupta and Gupta, 2017), SeCys methyltransferase (SMT) (Neuhierl and Boeck, 1996; Brummell et al., 2011; Chen et al., 2019) and SeCys lyase (SCL) (Pilon-Smits and Quinn, 2010). Because of this, most plants prevent excessive selenoamino acid accumulation to avoid metabolic impairments, especially those that may affect structural integrity and protein functions (Burnell, 1981; Brown and Shrift, 1982). The presence of Se in excess causes serious disruption at the metabolic level, including major changes in energy metabolism and ATP production, starch mobilization, and cell wall extension, which explains how Se causes a plant growth reduction (Ribeiro et al., 2016).

Selenoamino acids appear to be beneficial to growth in some conditions with an underlying influence on the oxidative protection networks in plants (Pilon-Smits and Quinn, 2010; Feng et al., 2013; Ahmad et al., 2016). Different strategies have been adopted to evaluate the role of Se in response to stress, including the application of Se as a seed priming treatment (Hasanuzzaman and Fujita, 2011; Nawaz et al., 2013; Hussain et al., 2016), soil fertilizer (Kumar et al., 2014; Khan et al., 2015; Atarodi et al., 2018), and foliar drench (Iqbal et al., 2015; Shahverdi et al., 2020). Treatment with Se at low concentrations is known to alleviate several stresses in plants, including those ones caused by drought (Hasanuzzaman and Fujita, 2011; Nawaz et al., 2013), heat (Iqbal et al., 2015), arsenic (Kumar et al., 2014), cadmium (Khan et al., 2015), low phosphorus (Jia et al., 2018), salinity (Shahverdi et al., 2020), as well as lead and aluminum (Feng et al., 2013). In addition to positive results in responding to several stresses, low concentrations of Se can also induce plant growth (Lehotai et al., 2012; Boldrin et al., 2016), via an effect on mitochondrial metabolism (Dimkovikj and Van Hoewyk, 2014) and molecular switches (Lehotai et al., 2012; Khan et al., 2015; Jia et al., 2018).

Concerning specific organs, several studies have demonstrated that this element delays fruit ripening (Zhu et al., 2017; Choudhary and Jain, 2018) and senescence (Pezzarossa et al., 2012, 2014), leading to reductions in postharvest losses. Its ability to alter these processes is related to increased glutathione peroxidase (GSH-Px) activity (Rayman, 2002; Hasanuzzaman et al., 2010; Feng et al., 2013), neutralization of oxidative stress through the inhibition of lipid peroxidation (Cartes et al., 2005), and ethylene biosynthesis downregulation (Pezzarossa et al., 2014). However, despite some studies had examined the effect of Se on postharvest vase life in cut flowers (Tognon et al., 2016; Lu et al., 2020), none of them investigated yet the relationship between Se and ethylene biosynthesis directly.

## Ethylene Metabolism and Its Inhibitors

As a simple gaseous hormone, ethylene acts in many fundamental processes in the plant’s life cycle, including regulation of leaf and root development, senescence, fruit ripening, and germination. Ethylene also acts in response to several abiotic stresses such as heat (Savada et al., 2017), heavy metals damage (Thao et al., 2015), salinity (Zhang et al., 2016; Silva et al., 2018), low soil pH (Brito et al., 2018; Ribeiro et al., 2018), and water deficiency (Dubois et al., 2017), triggering adaptive responses (Wang et al., 2002).

The complete elucidation of the ethylene biosynthetic pathway by Yang and Hoffman (1984) was a notable episode for the progress of studies of this hormone in higher plants. Ethylene is synthesized from carbons C3 and C4 of methionine (Met) through three key enzymatic reactions: (i) conversion of Met into *S*-adenosyl-L-methionine (SAM) by the enzyme SAM synthetase (SAMS); (ii) conversion of SAM to 1-aminocyclopropane-1-carboxylic acid (ACC) by the enzyme ACC synthase (ACS); and (iii) conversion of ACC to ethylene by the enzyme ACC oxidase (ACO). The 2nd step in this process, i.e., the formation of ACC from SAM is considered the rate-limiting step, since the formation of ethylene is strongly controlled by the ACS enzyme (Yang and Hoffman, 1984; Alonso and Ecker, 2001; Pattyn et al., 2020). The final conversion of ACC to ethylene is oxygen-dependent (Kende, 1993) and yields CO_2_ and cyanide as by-products. Once it is synthesized and perceived, the ethylene signaling pathway involves both positive and negative regulators, with the initial steps of signal transduction occurring at the endoplasmic reticulum membrane. The signal transduction involves ethylene receptors and transcription factors, with negative regulators inhibiting downstream responses via protein phosphorylation under the absence of ethylene (Azhar et al., 2019; Binder, 2020).

Ethylene biosynthesis/action inhibitors and ethylene removal technologies can mitigate premature senescence and abscission caused by exposure to exogenous or endogenous ethylene (Martínez-Romero et al., 2007). Interference in ethylene biosynthesis in ornamental plants can be achieved by blocking components of the ethylene synthesis pathway. Inhibition of the conversion of SAM to ACC by the compounds 1-aminoethoxyvinylglicine (AVG) and aminooxy acetic acid (AOA) effectively blocks the increase in ethylene production that accompanies senescence in a variety of ethylene sensitive flowers (Broun and Mayak, 1981; Serek and Andersen, 1993).

The inhibition of ethylene action is achieved by the use of antagonist molecules that bind to ethylene receptors, thus preventing downstream signaling. Among them, 2,5-norbornadiene (2,5-NBD) (Wang and Woodson, 1989), diazocyclopentadiene (DACP) (Blankenship and Sisler, 1993; Sisler et al., 1993; Serek et al., 1994), silver thiosulphate (Veen, 1979; Celikel and Reid, 2002), and 1-methyl cyclopropane (1-MCP) (Serek et al., 1995, 2006a; Sisler et al., 1999) are the most commonly studied and exploited. 1-MCP is the most commonly-used compound to control ethylene action during postharvest handling of fruits, flowers and vegetables commercially (Sisler and Serek, 1997). Its inhibitory mechanism is a result of competitive interaction with the ethylene receptors, since the ligand-binding site affinity is higher for 1-MCP than that of the gaseous hormone itself (Blankenship and Dole, 2006; Serek et al., 2006a). Nevertheless, it is thought that the development of new receptors recovers tissue sensitivity to ethylene in some plant materials, which can be treated with multiple applications of 1-MCP (Feng et al., 2004; Blankenship and Dole, 2006; In et al., 2013). On the other hand, 2,5-NBD also competes with ethylene for binding to ethylene receptors; however, high concentrations of ethylene can overcome the inhibitory effect of 2,5-NBD (Sisler and Yang, 1984). Moreover, 2,5-NBD is limitedly useful commercially as an ethylene inhibitor since it requires continuous exposure to be effective, and presents a strong and disagreeable odor (Sisler et al., 1990). Similarly, DACP is unlikely to be used commercially due to its instability and hazardous characteristics (Serek et al., 2006b). Finally, silver ions (Ag^+^) may also block ethylene action, perhaps by replacing the metal component in the receptor. However, commercial use of silver is limited due to its heavy metal toxicity (Atta-Aly et al., 1987). Furthermore, the use of solutions containing silver íon by florists has raised environmental concerns, mostly regarding disposal issues (Sisler and Serek, 1997).

## Model for Se-Induced Downregulation of Ethylene Biosynthesis in Cut Flower

Recently, a direct interaction between Se and ethylene was elegantly demonstrated in experiments involving cadmium stress alleviation in wheat (Iqbal et al., 2015), tomato fruit ripening (Zhu et al., 2017), and control of primary root growth in the rice system (Malheiros et al., 2019). These independent but complementary studies generated shreds of evidence that such responses were a consequence of an ethylene biosynthesis downregulation induced by Se. In close agreement with this, Se was also reported to improve minimally processed vegetable life span through ethylene depletion (Malorgio et al., 2009).

**FIGURE 1:**
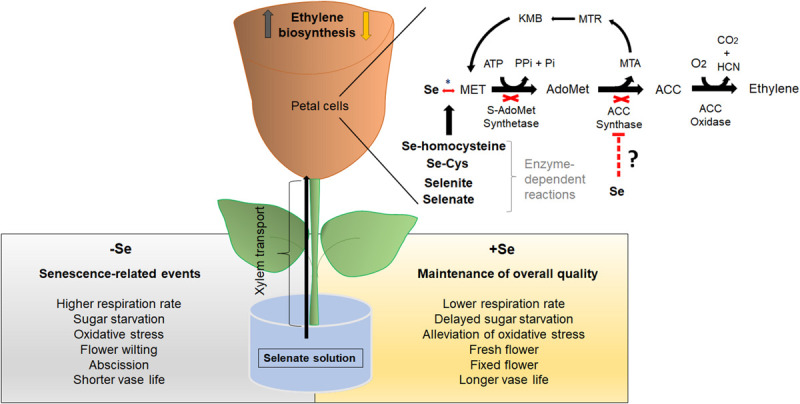
Model for Se-induced ethylene biosynthesis downregulation in a cut flower system. Firstly, Se is provided as selenate – the main transported form in the xylem. In petal cells, selenate is converted to selenite and subsequently to Se-homocysteine by a series of enzymatic reactions. In a third stage, Se-homocysteine forms SeMet, reducing free Met levels toward to biosynthesis ethylene pathway. This event affects subsequently SAMS and ACS activities by reducing substrate (Met) availability to sustain ethylene biosynthesis pathway. Finally, the decreased ethylene biosynthesis may lead to the maintenance of overall quality on the postharvest life of cut flowers. Ethylene biosynthesis pathway: ACC, 1-aminocyclopropane-1-carboxylate; ACS, ACC synthase; MTR, *S*-methyl-5-thio-D-ribose; MTA, *S-*methyl-5′-this adenosine; KMB, 2-keto-4-methylthiobutyrate; Se, Selenium; SAM, *S-*adenosylmethionine; SAM synthetase, SAMS; HCN, Hydrogen cyanide; CO_2_, Carbon dioxide; O_2_, Oxygen; Met, Methionine; Pi, inorganic phosphate; and PPi, inorganic pyrophosphate. Se metabolism: Se, Selenium; SeCys, selenocysteine; SeMet, selenomethionine. *Enzymatic reaction.

In this review, we propose the action of Se on ethylene biosynthesis in a cut flower model system through selenate (Figure 1) – the main form of Se to be transported in the xylem (Asher et al., 1977; Terry et al., 2000). The first step of Se metabolism in the cells involves the reduction of selenate to selenite under the sequential action of two enzymes, ATP sulfurylase (ATPS) and APS reductase (APR) (Shaw and Anderson, 1972; Sors et al., 2005; Pilon-Smits and Quinn, 2010; Quinn et al., 2011; Gupta and Gupta, 2017). ATPS catalyzes the hydrolysis of ATP, coupling ATP to selenate to form adenosine phosphoselenate (APSe), being subsequently reduced to selenite by APR (Sors et al., 2005; Pilon-Smits and Quinn, 2010). Both enzymes are present in the cytosol and plastids, but this process occurs primarily in the plastids, as observed in S metabolism (Kolosova et al., 2001). The reduction from selenite to selenide is also carried out in an enzyme-independent way by reduced glutathione (GSH) (Terry et al., 2000; Wallenberg et al., 2010). In the presence of the cysteine synthase (CS) enzyme, selenide can be converted into SeCys by coupling with *O*-acetylserine (OAS) (Ng and Anderson, 1978).

Selenocysteine may be incorporated into proteins, thereby impairing their activities (Burnell, 1981; Brown and Shrift, 1982; Terry et al., 2000). On the other hand, SeCys can be also transferred to Met, forming MeSeCys by selenocysteine methyltransferase (SMT) (Sors et al., 2005; Gupta and Gupta, 2017). In this case, a critical point arises since Met is shared with the ethylene biosynthesis pathway (Figure 1). For such convergences, it has been suggested that SeMet reduces free Met, which in turn diminishes internal ethylene levels by limiting the substrate for SAMS and ACS activities (Konze and Kende, 1979; Malorgio et al., 2009; Iqbal et al., 2013, 2015). The improvement of cut flowers vase life by manipulating ethylene biosynthesis has been addressed in several previous studies (Baker et al., 1977; Wang et al., 1977; Reid and Wu, 1992; Zeng et al., 2012). Kosugi et al. (2002), for instance, demonstrated that the suppression of ethylene biosynthesis in the *ACO* antisense line prolonged the vase life of carnation by 1.6-fold, as compared to its counterpart. In our proposed model (Figure 1), we hypothesize that Se diminishes ethylene levels in cut flowers by reducing the presence of free precursor Met to sustain ethylene biosynthesis, leading to extended postharvest life in ethylene-sensitive species.

## Downstream Responses Associated With Se Use in Vase Solution

Senescence is a complex, critical, and coordinated event that determines the longevity of cut flowers (Wu et al., 2017; Aalifar et al., 2020). The final phase of flower vase life, for instance, is characterized by time-dependent petal wilting, flower withering (Su et al., 2019), and flower or petal abscission (Van Doorn, 2001). Some flowers usually show symptoms of color change and desiccation of petals before abscission (Ma et al., 2005; Shibuya et al., 2016).

Ethylene is one of the most important hormones involved in the regulation of flower senescence (Ma et al., 2018; Wang et al., 2020) and elicits responses at concentrations as low as 0.1 μL L^–1^ in highly sensitive flowers (Macnish et al., 2011). Sensitivity to ethylene differs between species and cultivars (Macnish et al., 2010; Costa and Finger, 2016; Wu et al., 2017). In ethylene-sensitive species, ethylene induces endogenous and autocatalytic ethylene biosynthesis that triggers petal and flower senescence. Ethylene causes petal and flower wilting during senescence by inhibiting cell expansion through the regulation of aquaporins (Ma et al., 2008), proteins that promote water transport through biological membranes (Xue et al., 2020). This causes subsequently a negative water balance, a key limiting event in the vase life of cut flowers (Van Meeteren and Aliniaeifard, 2016).

High rates of respiration are also one of the main causes of short vase life in cut flowers (Jones et al., 2009). Ethylene is known to induce respiratory activity, thereby depleting carbohydrates levels (Gonzalez-Candelas et al., 2010; John-Karuppiah and Burns, 2010). On the other hand, ethylene is also involved with flower abscission by triggering abscission zone formation (Van Doorn, 2002) and by oxidative stress promoted by ROS, including the overproduction of superoxide anion (O_2_^–^) and hydrogen peroxide (H_2_O_2_) (Rogers and Munné-Bosch, 2016; Ren et al., 2017; Jędrzejuk et al., 2018; Bayanati et al., 2019).

Therefore, we suggest that Se increases vase life directly by downregulating ethylene synthesis and indirectly by reducing flower senescence-related events, such as respiration rate, sugar starvation, petal and flower wilting and abscission, and oxidative stress (Figure 1).

## Conclusion and Broader Perspectives

Herein, we have proposed a new model of interaction between Se metabolism and ethylene biosynthesis, and pointed out the positive effects of this event on the postharvest life of cut flowers. We believe the use of Se can provide a commercially viable and environmentally friendly alternative to current methods applied to ethylene-sensitive cut flowers. Practical aspects such as doses and standard use methods should be further investigated for each species under study.

## Author Contributions

LC, LL, and VN conceptualized and organized all this manuscript. LC, LL, VN, and MS contributed in survey and writing for selenium metabolism. VN, FA, CF, and TS performed a survey and writing for ethylene metabolism and postharvest quality of flowers. KF and FF supervised all the surveys and writing. All authors equally contributed to the development of the article’s theoretical framework and approved the submitted version.

## Conflict of Interest

The authors declare that the research was conducted in the absence of any commercial or financial relationships that could be construed as a potential conflict of interest.

## References

[B1] Aalifar M. Aliniaeifard S. Arab M. Mehrjerdi M. Serek M. (2020). Blue light postpones senescence of carnation flowers through regulation of ethylene and abscisic acid pathway-related genes. *Plant Physiol. Biochem.* 151 103–112. 10.1016/j.plaphy.2020.03.018 32208322

[B2] Ahmad R. Waraich E. A. Nawaz F. Ashraf M. Y. Khalid M. (2016). Selenium (Se) improves drought tolerance in crop plants- a myth or fact? *J. Sci. Food Agric.* 96 372–380. 10.1002/jsfa.7231 25906838

[B3] Alonso J. M. Ecker J. R. (2001). The ethylene pathway: a paradigm for plant hormone signalling and interaction. *Sci. Signal.* 70:re1 10.1126/stke.2001.70.re1 11752640

[B4] Asher C. J. Butler G. W. Peterson P. J. (1977). Selenium transport in root systems of tomato. *J. Exp. Bot.* 28 279–291. 10.1093/jxb/28.2.279

[B5] Atarodi B. Fotovat A. Khorassani R. Keshavarz P. Hammami H. (2018). Interaction of selenium and cadmium in wheat at different salinities. *Toxicol. Environ. Chem.* 100 348–360. 10.1080/02772248.2018.1524472

[B6] Atta-Aly M. A. Saltveit M. E. Hobson G. E. (1987). Effect of silver ions on ethylene biosynthesis by tomato fruit tissue. *Plant Physiol.* 83 44–48. 10.1104/pp.83.1.44 16665213 PMC1056296

[B7] Azhar B. J. Zulfiqar A. Shakeel S. N. Schaller G. E. (2019). Amplification and adaptation in the ethylene signaling pathway. *Small Methods* 4:1900452 10.1002/smtd.201900452

[B8] Baker J. E. Wang C. Y. Lieberman M. Hardenburg R. (1977). Delay of senescence in carnations by rhizobitoxine analog and sodium benzoate. *HortScience* 12 38–39.

[B9] Barry C. S. Giovannoni J. J. (2007). Ethylene and fruit ripening. *J. Plant Growth Regul.* 26 143–159. 10.1007/s00344-007-9002-y

[B10] Bayanati M. Tehranifar A. Razavi K. Nemati S. H. Lohrasebi T. Ahmadi N. (2019). Expression patterns analysis of SOD genes in responses to ethylene-induced oxidative stress in rose (Rosa hybrida) during flower development. *S. Afr. J. Bot.* 127 265–270. 10.1016/j.sajb.2019.09.009

[B11] Binder B. M. (2020). Ethylene signaling in plants. *J. Biol. Chem.* 295 7710–7725. 10.1074/jbc.REV120.010854 32332098 PMC7261785

[B12] Blankenship S. M. Dole J. M. (2006). 1-Methylcyclopropene: a review. *Postharvest Biol. Technol.* 28 1–25. 10.1016/S0925-5214(02)00246-6

[B13] Blankenship S. M. Sisler E. C. (1993). Response of apples to diazocyclopentadiene inhibition of ethylene binding. *Postharvest Biol. Technol.* 3 95–101. 10.1016/0925-5214(93)90001-J

[B14] Boldrin P. F. de Figueiredo M. A. Yang Y. Luo H. Giri S. Hart J. J. (2016). Selenium promotes sulfur accumulation and plant growth in wheat (*Triticum aestivum*). *Physiol. Plant.* 158 80–91. 10.1111/ppl.12465 27152969

[B15] Brito F. A. L. Costa L. C. Gasparini K. Pimenta T. M. Araujo W. L. Zsögön A. (2018). Low soil pH modulates ethylene biosynthesis and germination response of Stylosanthes humilis seeds. *Plant Signal Behav.* 13:e1460186 10.1080/15592324.2018.1460186 PMC6103276 29746797

[B16] Broun R. Mayak S. (1981). Aminooxyacetic acid as an inhibitor of ethylene synthesis and senescence in carnation flowers. *Sci. Hortic.* 15 277–282. 10.1016/0304-4238(81)90038-8

[B17] Brown T. A. Shrift A. (1982). Selenium: toxicity and tolerance in higher plants. *Biol. Rev.* 57 59–84. 10.1111/j.1469-185X.1982.tb00364.x

[B18] Brummell D. A. Watson L. M. Pathirana R. Joyce N. I. West P. J. Hunter D. A. (2011). Biofortification of tomato (*Solanum lycopersicum*) fruit with the anticancer compound methylselenocysteine using a selenocysteine methyltransferase from a selenium hyperaccumulator. *J. Agric. Food Chem.* 59 10987–10994. 10.1021/jf202583f 21942920

[B19] Burnell J. N. (1981). Selenium metabolism in *Neptunia amplexicaulis*. *Plant Physiol.* 67 316–324. 10.1104/pp.67.2.316 16661667 PMC425675

[B20] Cartes P. Gianfreda L. Mora M. L. (2005). Uptake of selenium and its antioxidant activity in ryegrass when applied as selenate and selenite forms. *Plant Soil* 276 359–367. 10.1007/s11104-005-5691-9

[B21] Celikel F. G. Reid M. S. (2002). Postharvest handling of stock (*Matthiola incana*). *HortScience* 37 144–147. 10.21273/HORTSCI.37.1.144

[B22] Chen M. Zeng L. Luo X. Mehboob M. Z. Tegenbaiyin A. O. Lang M. (2019). Identification and functional characterization of a novel selenocysteine methyltransferase from *Brassica juncea* L. *J. Exp. Bot.* 70 6401–6416. 10.1093/jxb/erz390 31504785

[B23] Choudhary P. Jain V. (2018). Effect of post-harvest treatments of selenium on physico-chemical quality in guava (*Psidium guajava* L.) fruit. *Hortic. Int. J.* 2 41–44. 10.15406/hij.2018.02.00024

[B24] Cochran A. T. Bauer J. Metcalf J. L. Lovecka P. Sura-de Jong M. Warris S. (2018). Plant selenium hyperaccumulation affects rhizosphere: enhanced species richness and altered species composition. *Phytobiomes J.* 2 82–91. 10.1094/pbiomes-12-17-0051-R

[B25] Corbineau F. Xia Q. Bailly C. El-Maarouf-Bouteau H. (2014). Ethylene, a key factor in the regulation of seed dormancy. *Front. Plant Sci.* 10:539 10.3389/fpls.2014.00539 PMC4193209 25346747

[B26] Costa L. C. Finger F. L. (2016). Flower opening and vase life of gladiolus cultivars: the sensitivity to ethylene and the carbohydrate content. *Ornam. Hortic.* 22 147–153. 10.14295/oh.v22i2.901

[B27] Daniels L. A. (1996). Selenium metabolism and bioavailability. *Biol. Trace Elem. Res.* 54 185–199. 10.1007/BF02784430 8909692

[B28] De Martinis D. Koyama T. Chang C. (2015). Ethylene is all around. *Front. Plant Sci.* 6:76 10.3389/fpls.2015.00076 PMC4325880 25729386

[B29] Dimkovikj A. Van Hoewyk D. (2014). Selenite activates the alternative oxidase pathway and alters primary metabolism in *Brassica napus* roots: evidence of a mitochondrial stress response. *BMC Plant Biol.* 14:259 10.1186/s12870-014-0259-6 PMC4189625 25267309

[B30] Dubois M. Claeys H. Van den Broeck L. Inzé D. (2017). Time of day determines *Arabidopsis transcriptome* and growth dynamics under mild drought. *Plant Cell Environ.* 40 180–189. 10.1111/pce.12809 27479938

[B31] Feng R. W. Wei C. Y. Tu S. X. (2013). The roles of selenium in protecting plants against abiotic stresses. *Environ. Exp. Bot.* 87 58–68. 10.1016/j.envexpbot.2012.09.002

[B32] Feng X. Q. Apelbaum A. Sisler E. C. Goren R. (2004). Control of ethylene activity in various plant systems by structural analogues of 1-methylcyclopropene. *Plant Growth Regul.* 42 29–38.

[B33] Fordyce F. M. (2005). “Selenium deficiency and toxicity in the environment,” in *Essentials of Medical Geology*, ed. Selinus O. (Dordrecht: Springer), 373–415.

[B34] Freeman J. L. Tamaoki M. Stushnoff C. (2010). Molecular mechanisms of selenium tolerance and hyperaccumulation in *Stanleya pinnata*. *Plant Physiol.* 153 1630–1652. 10.1104/pp.110.156570 20498337 PMC2923907

[B35] Galeas M. L. Zhang L. H. Freeman J. L. Wegner M. Pilon-Smits E. A. H. (2007). Seasonal fluctuations of selenium and sulfur accumulation in selenium hyperaccumulators and related non-accumulators. *New Phytol.* 173 517–525. 10.1111/j.1469-8137.2006.01943.x 17244046

[B36] Giovannoni J. J. (2001). Molecular biology of fruit maturation and ripening. *Annu. Rev. Plant Physiol. Plant Mol. Biol.* 52 725–749. 10.1146/annurev.arplant.52.1.725 11337414

[B37] Gonzalez-Candelas L. Alamar S. Sanchez-Torres P. Zacarias L. Marcos J. F. (2010). A transcriptomic approach highlights induction of secondary metabolism in citrus fruit in response to Penicillium digitatum infection. *BMC Plant Biol.* 10:194 10.1186/1471-2229-10-194 PMC2956543 20807411

[B38] Gupta M. Gupta S. (2017). An overview of selenium uptake, metabolism, and toxicity in plants. *Front. Plant Sci.* 7:2074 10.3389/fpls.2016.02074 PMC5225104 28123395

[B39] Hajiboland R. Rahmat S. Zeinalzadeh N. Farsad-Akhtar N. Hosseinpour-Feizi M.-A. (2019). Senescence is delayed by selenium in oilseed rape plants. *J. Trace Elem. Med. Biol.* 55 96–106. 31345373 10.1016/j.jtemb.2019.06.005

[B40] Hasanuzzaman M. Fujita M. (2011). Selenium pretreatment upregulates the antioxidant defense and methylglyoxal detoxification system and confers enhanced tolerance to drought stress in rapeseed seedlings. *Biol. Trace Elem. Res.* 143 1758–1776. 10.1007/s12011-011-8998-9 21347652

[B41] Hasanuzzaman M. Hossain M. A. Fujita M. (2010). Selenium in higher plants: physiological role, antioxidant metabolism and abiotic stress tolerance. *J. Plant Sci.* 4 354–375. 10.3923/jps.2010.354.375

[B42] Haug A. Graham R. D. Christopherson O. A. Lyons G. H. (2008). How to use the world’s scarce selenium resources efficiently to increase the selenium concentration in food. *Microb. Ecol. Health Dis.* 19 209–228. 10.1080/08910600701698986 PMC2556185 18833333

[B43] Hawrylak-Nowak B. Dreslerb S. Wójcik M. (2014). Selenium affects physiological parameters and phytochelatins accumulation in cucumber (*Cucumis sativus* L.) plants grown under cadmium exposure. *Sci. Hort.* 172 10–18. 10.1016/j.scienta.2014.03.040

[B44] Huang W. N. Liu H. K. Zhang H. H. Chen Z. Guo Y. D. Kang Y. F. (2013). Ethylene-induced changes in lignification and cell wall-degrading enzymes in the roots of mungbean (*Vigna radiata*) sprouts. *Plant Physiol. Biochem.* 73 412–419. 10.1016/j.plaphy.2013.10.020 24239576

[B45] Hussain S. Khan F. Cao W. Wu L. Geng M. (2016). Seed priming alters the production and detoxification of reactive oxygen intermediates in rice seedlings grown under sub-optimal temperature and nutrient supply. *Front. Plant Sci.* 7:439 10.3389/fpls.2016.00439 PMC4820636 27092157

[B46] In B. C. Strable J. Binder B. M. Falbel T. G. Patterson S. E. (2013). Morphological and molecular characterization of ethylene binding incarnations. *Post. Harvest Biol. Technol.* 86 272–279. 10.1016/j.postharvbio.2013.07.007

[B47] Iqbal M. Hussain I. Liaqat H. Arslan Ashraf M. Rasheed R. Ur Rehman A. (2015). Exogenously applied selenium reduces oxidative stress and induces heat tolerance in spring wheat. *Plant Physiol. Biochem.* 94 95–103. 10.1016/j.plaphy.2015.05.012 26057700

[B48] Iqbal N. Trivellini A. Masood A. Ferrante A. Khan N. A. (2013). Current understanding on ethylene signaling in plants: the influence of nutrient availability. *Plant Physiol. Biochem.* 73 128–138. 10.1016/j.plaphy.2013.09.011 24095919

[B49] Ivanchenko M. G. Muday G. K. Dubrovsky J. G. (2008). Ethylene-auxin interactions regulate lateral root initiation and emergence in *Arabidopsis thaliana*. *Plant J.* 55 335–347. 10.1111/j.1365-313X.2008.03528.x 18435826

[B50] Iwahori S. Lyons J. M. Smith O. E. (1970). Sex expression in cucumber plants as affected by 2-chloroethylphosphonic acid, ethylene, and growth regulators. *Plant Physiol.* 46 412–415. 10.1104/pp.46.3.412 16657476 PMC396605

[B51] Jędrzejuk A. Rabiza-Świder J. Skutnik E. Łukaszewska A. (2018). Growing conditions and preservatives affect longevity, soluble protein, H2O2 and MDA contents, activity of antioxidant enzymes and DNA degradation in cut lilacs. *Sci. Hortic.* 228 122–131. 10.1016/j.scienta.2017.10.026

[B52] Jia H. Song Z. Wu F. Ma M. Li Y. Han D. (2018). Low selenium increases the auxin concentration and enhances tolerance to low phosphorous stress in tobacco. *Environ. Exp. Bot.* 153 127–134. 10.1016/j.envexpbot.2018.05.017

[B53] John-Karuppiah K. Burns J. K. (2010). Degreening behavior in ‘Fallglo’ and ‘Lee×Orlando’ is correlated with differential expression of ethylene signaling and biosynthesis genes. *Postharvest Biol. Technol.* 58 185–193. 10.1016/j.postharvbio.2010.07.013

[B54] Jones M. L. Stead A. D. Clark D. G. (2009). “Petunia flower senescence,” in *Petunia*, eds Gerats T. Strommer J. (New York, NY: Springer), 301–324.

[B55] Kende H. (1993). Ethylene biosynthesis. *Annu. Rev. Plant Physiol. Plant Mol. Biol.* 44 283–307. 10.1146/annurev.pp.44.060193.001435

[B56] Khan M. I. R. Asgher M. Khan N. A. (2014). Alleviation of salt-induced photosynthesis and growth inhibition by salicylic acid involves glycinebetaine and ethylene in mung bean (*Vigna radiata* L.). *Plant Physiol. Biochem.* 80 67–74. 10.1016/j.plaphy.2014.03.026 24727790

[B57] Khan M. I. R. Nazir F. Asgher M. Per T. S. Khan N. A. (2015). Selenium and sulfur influence ethylene formation and alleviate cadmium-induced oxidative stress by improving proline and glutathione production in wheat. *J. Plant Physiol.* 173 9–18. 10.1016/j.jplph.2014.09.011 25462073

[B58] Khan N. A. Khan M. I. R. Asgher M. Fatma M. Masood A. Syeed S. (2014). Salinity tolerance in plants: revisiting the role of sulfur metabolites. *J. Plant Biochem. Physiol.* 2:120 10.4172/2329-9029.1000120

[B59] Kim H. J. Hong S. H. Kim Y. W. Lee I. H. Jun J. H. Phee B. K. (2014). Gene regulatory cascade of senescence-associated NAC transcription factors activated by ETHYLENE-INSENSITIVE2-mediated leaf senescence signalling in *Arabidopsis*. *J. Exp. Bot.* 65 4023–4036. 10.1093/jxb/eru112 24659488 PMC4106440

[B60] Kolosova N. Sherman D. Karlson D. Dudareva N. (2001). Cellular and subcellular localization of S-adenosyl-L-methionine:benzoic acid carboxyl methyltransferase, the enzyme responsible for biosynthesis of the volatile ester methylbenzoate in snapdragon flowers. *Plant Physiol.* 126 956–964. 10.1104/pp.126.3.956 11457946 PMC116452

[B61] Konze J. R. Kende H. (1979). Interactions of methionine and selenomethionine with methionine adenosyltransferase and ethylene-generating systems. *Plant Physiol.* 63 507–510. 10.1104/pp.63.3.507 16660757 PMC542860

[B62] Kosugi Y. Waki K. Iwazaki Y. Tsuruno N. Mochizuki A. Yoshioka T. (2002). Senescence and gene expression of transgenic non-ethylene-producing carnation flowers. *J. Jpn. Soc. Hortic. Sci.* 71 638–642.

[B63] Kumar A. Singh R. P. Singh P. K. Awasthi S. Chakrabarty D. Trivedi P. K. (2014). Selenium ameliorates arsenic induced oxidative stress through modulation of antioxidant enzymes and thiols in rice (*Oryza sativa* L.). *Ecotoxicology* 23 1153–1163. 10.1007/s10646-014-1257-z 24985886

[B64] Lapaz A. M. Santos L. F. M. Yoshida C. H. P. Heinrichs R. Campos M. Reis A. R. (2019). Physiological and toxic effects of selenium on seed germination of cowpea seedlings. *Bragantia.* 4 1–11. 10.1590/1678-4499.20190114

[B65] Lehotai N. Kolbert Z. Peto A. Feigl G. Ordog A. Kumar D. (2012). Selenite-induced hormonal and signalling mechanisms during root growth of *Arabidopsis thaliana* L. *J. Exp. Bot.* 63 5677–5687. 10.1093/jxb/err313 22988013

[B66] Liang Y. Yang S. U. Ling L. I. Xin H. Panhwar F. Zheng T. (2019). Quick selenium accumulation in the selenium-rich rice and its physiological responses in changing selenium environments. *BMC Plant Biol.* 19:559 10.1186/s12870-019-2163-6 PMC6918634 31847801

[B67] Lim P. O. Kim H. J. Nam H. G. (2007). Leaf senescence. *Annu. Rev. Plant Biol.* 58 115–136. 10.1146/annurev.arplant.57.032905.105316 17177638

[B68] Lima J. E. Benedito V. A. Figueira A. Peres L. E. P. (2009). Callus, shoot and hairy root formation in vitro as affected by the sensitivity to auxin and ethylene in tomato mutants. *Plant Cell Rep.* 28 1169–1177. 10.1007/s00299-009-0718-y 19484241

[B69] Lin Y. Yang L. Paul M. Zu Y. Tang Z. (2013). Ethylene promotes germination of Arabidopsis seed under salinity by decreasing reactive oxygen species: Evidence for the involvement of nitric oxide simulated by sodium nitroprusside. *Plant Physiol. Biochem.* 73 211–218. 10.1016/j.plaphy.2013.10.003 24148906

[B70] Lobanov A. V. Hatfield D. L. Gladyshev V. N. (2009). Eukaryotic selenoproteins and selenoproteomes. *BBA Gen. Subjects* 1790 1424–1428. 10.1016/j.bbagen.2009.05.014 PMC3471088 19477234

[B71] Lu N. Wu L. Shi M. (2020). Selenium enhances the vase life of Lilium longiflorum cut flower by regulating postharvest physiological characteristics. *Sci. Hortic.* 264:109172 10.1016/j.scienta.2019.109172

[B72] Ma N. Cai L. Wangjin L. Tan H. Gao J. (2005). Exogenous Ethylene influences flower opening of cut roses (Rosa hybrida) by regulating the genes encoding ethylene biosynthesis enzymes. *Sci. Shina Ser. B* 48:434 10.1360/062004-37 16315594

[B73] Ma N. Ma C. Liu Y. Shahid M. Wang C. Gao J. (2018). Petal senescence: a hormone view. *J. Exp. Bot.* 69 719–732. 10.1093/jxb/ery009 29425359

[B74] Ma N. Xue J. Li Y. Liu X. Dai F. Jia (2008). Rh-PIP2;1, a rose aquaporin gene, is involved in ethylene-regulated petal expansion. *Plant Physiol.* 148 894–907. 10.1104/pp.108.120154 18715962 PMC2556823

[B75] Macnish A. J. Leonard R. T. Borda A. M. Nell T. A. (2010). Genotypic variation in the postharvest performance and ethylene sensitivity of cut rose flowers. *Hortscience.* 45 790–796. 10.21273/hortsci.45.5.790

[B76] Macnish A. J. Leonard R. T. Nell T. A. (2011). Sensitivity of potted foliage plant genotypes to ethylene and 1-methylcyclopropene. *Hortscience* 46 1127–1131. 10.21273/hortsci.46.8.1127

[B77] Malheiros R. S. P. Costa L. C. Ávila R. T. Pimenta T. M. Teixeira L. S. Brito F. A. L. (2019). Selenium downregulates auxin and ethylene biosynthesis in rice seedlings to modify primary metabolism and root architecture. *Planta* 250 333–345. 10.1007/s00425-019-03175-6 31030327

[B78] Malorgio F. Diaz K. E. Ferrante A. Mensuali-Sodi A. Pezzarossa B. (2009). Effects of selenium addition on minimally processed leafy vegetables grown in a floating system. *J. Sci. Food Agric.* 89 2243–2251. 10.1002/jsfa.3714

[B79] Martínez-Romero D. Bailén G. Serrano M. Guillén F. Valverde J. M. Zapata P. (2007). Tools to maintain postharvest fruit and vegetable quality through the inhibition of ethylene action: a review. *Crit. Rev. Food Sci. Nutr.* 47 543–560. 10.1080/10408390600846390 17653980

[B80] Miransari M. Smith D. L. (2014). Plant hormones and seed germination. *Environ. Exp. Bot.* 99 110–121. 10.1016/j.envexpbot.2013.11.005

[B81] Morgan P. W. Drew M. C. (1997). Ethylene and plant responses to stress. *Physiol. Plant* 100 620–630. 10.1034/j.1399-3054.1997.1000325.x

[B82] Nancharaiah Y. V. Lens P. N. (2015). Ecology and biotechnology of selenium-respiring bacteria. *Microbiol. Mol. Biol. R.* 79 61–80. 10.1128/MMBR.00037-14 PMC4402961 25631289

[B83] Nawaz F. Ashraf M. Y. Ahmad R. Waraich E. A. (2013). Selenium (Se) seed priming induced growth and biochemical changes in wheat under water deficit conditions. *Biol. Trace Elem. Res.* 151 284–293. 10.1007/s12011-012-9556-9 23197374

[B84] Neal R. H. Sposito G. Holtzclaw K. M. Traina S.l (1987). Selenite adsorption on alluvial soils. I. Soil composition and pH effects. *Soil Sci. Soc. Am. J.* 51 1161–1165. 10.2136/sssaj1987.03615995005100050012x

[B85] Neuhierl B. Boeck A. (1996). On the mechanism of selenium tolerance in selenium accumulating plants: purification and characterization of a specific selenocysteine methyltransferase from cultured cells of *Astragalus bisulcatus*. *Eur. J. Biochem.* 239 235–238. 10.1111/j.1432-1033.1996.0235u.x 8706715

[B86] Ng B. H. Anderson J. W. (1978). Synthesis of selenocysteine by cysteine synthases from selenium accumulator and non-accumulator plants. *Phytochemistry* 17 2069–2074. 10.1016/S0031-9422(00)89282-1

[B87] O’Neill S. D. (1997). Pollination regulation of flower development. *Annu. Rev. Plant Physiol. Plant Mol. Biol.* 48 547–574. 10.1146/annurev.arplant.48.1.547 15012274

[B88] Pattyn J. Vaughan−Hirsch J. Van de Poel B. (2020). The regulation of ethylene biosynthesis: a complex multilevel control circuitry. *New Phytol.* 10.1111/nph.16873 [Epub ahead of print]. PMC7820975 32790878

[B89] Pezzarossa B. Remorini D. Gentile M. L. (2012). Effects of foliar and fruit addition of sodium selenate on selenium accumulation and fruit quality. *J. Sci. Food Agric.* 92 781–786. 10.1002/jsfa.4644 21953507

[B90] Pezzarossa B. Rosellini I. Borghesi E. Tonutti P. Malorgio F. (2014). Effects of Se-enrichment on yield, fruit composition and ripening of tomato (*Solanum lycopersicum*) plants grown in hydroponics. *Sci. Hortic.* 65 106–110. 10.1016/j.scienta.2013.10.029

[B91] Pilon-Smits E. A. H. Quinn C. F. (2010). “Selenium metabolism in plants,” in *Cell Biology of Metals and Nutrients. Plant Cell Monographs*, eds Hell R. Mendel R. R. (Berlin: Springer), 225–241.

[B92] Quinn C. F. Prins C. N. Freeman J. L. Gross A. M. Hantzis L. J. Reynolds R. J. B. (2011). Selenium accumulation in flowers and its effects on pollination. *New Phytol.* 192 727–737. 10.1111/j.1469-8137.2011.03832.x 21793829

[B93] Rayman M. P. (2002). The argument for increasing selenium intake. *Proc. Nutr. Soc.* 2 203–215. 10.1079/PNS2002153 12133202

[B94] Reid M. S. Wu M. J. (1992). Ethylene and flower senescence. *Plant Growth Regul.* 11 37–43.

[B95] Ren P. J. Jin X. Liao W. B. Wang M. Niu L. J. Li X. P. (2017). Effect of hydrogen-rich water on vase life and quality in cut lily and rose flowers. *Hortic. Environ. Biotechnol.* 58 576–584. 10.1007/s13580-017-0043-2

[B96] Ribeiro D. M. Silva Júnior D. D. Cardoso F. B. Martins A. O. Silva W. A. Nascimento V. L. (2016). Growth inhibition by selenium is associated with changes inprimary metabolism and nutrient levels in *Arabidopsis thaliana*. *Plant Cell Environ.* 39 2235–2246. 10.1111/pce.12783 27342381

[B97] Ribeiro R. P. Costa L. C. Medina E. F. Araújo W. L. Zsögön A. Ribeiro D. M. (2018). Ethylene coordinates seed germination behavior in response to low soil pH in *Stylosanthes humilis*. *Plant Soil.* 425 87–100. 10.1007/s11104-018-3572-2 PMC6103276 29746797

[B98] Rogers H. Munné-Bosch S. (2016). Production and scavenging of reactive oxygen species and redox signaling during leaf and flower senescence: similar but different. *Plant Physiol.* 171 1560–1568. 10.1104/pp.16.00163 27208233 PMC4936548

[B99] Saidi I. Chtourou Y. Djebali W. (2014). Selenium alleviates cadmium toxicity by preven-ting oxidative stress in sunflower (*Helianthus annuus*) seedlings. *J. Plant Physiol.* 171 85–91. 10.1016/j.jplph.2013.09.024 24484961

[B100] Salman-Minkov A. Levi A. Wolf S. Trebitsh T. (2008). ACC synthase genes are polymorphic in watermelon (Citrullus spp.) and differentially expressed in flowers and in response to auxin and gibberellin. *Plant Cell Physiol.* 49 740–750. 10.1093/pcp/pcn045 18367517

[B101] Sattar A. Cheema M. A. Sher A. Ijaz M. Ul-Allah S. Nawaz A. (2019). Physiological and biochemical attributes of bread wheat (*Triticum aestivum* L.) seedlings are influenced by foliar application of silicon and selenium under water deficit. *Acta Physiol. Plant* 41:146 10.1007/s11738-019-2938-2

[B102] Savada R. P. Ozga J. A. Jayasinghege C. P. A. Waduthanthri K. D. Reinecke D. M. (2017). Heat stress differentially modifies ethylene biosynthesis and signaling in pea floral and fruit tissues. *Plant Mol. Biol.* 95 313–331. 10.1007/s11103-017-0653-1 28861701

[B103] Scariot V. Paradiso R. Rogers H. De Pascale S. (2014). Ethylene control in cut flowers: classical and innovative approaches. *Postharvest Biol. Technol.* 97 83–92. 10.1016/j.postharvbio.2014.06.010

[B104] Serek M. Andersen A. S. (1993). AOA and BA influence on floral development and longevity of potted ‘Victory Parade’ miniature rose. *HortScience* 28 1039–1040. 10.21273/HORTSCI.28.10.1039

[B105] Serek M. Sisler E. C. Frello S. Sriskandarajah S. (2006b). Postharvest technologies for extending the shelf life of ornamental crops. *Int. J. Postharvest Technol. Inn.* 1 69–75. 10.1504/IJPTI.2006.009184

[B106] Serek M. Sisler E. C. Reid M. S. (1994). Novel gaseous ethylene binding inhibitor prevents ethylene effects in potted flowering plants. *J. Am. Hort. Sci.* 119 1230–1233. 10.21273/JASHS.119.6.1230

[B107] Serek M. Sisler E. C. Reid M. S. (1995). 1-Methylcyclopropene, a novel gaseous inhibitor of ethylene action, improves the vase life of fruits, cut flowers and potted plants. *Acta Hort.* 394 337–346. 10.17660/ActaHortic.1995.394.37

[B108] Serek M. Woltering E. J. Sisler E. C. Frello S. Sriskandarajah S. (2006a). Controlling ethylene responses in flowers at the receptor level. *Biotechnol. Adv.* 24 368–381. 10.1016/j.biotechadv.2006.01.007 16584864

[B109] Shahverdi M. A. Omidi H. Damalas C. A. (2020). Foliar fertilization with micronutrients improves Stevia rebaudiana tolerance to salinity stress by improving root characteristics. *Braz. J. Bot.* 43 55–65. 10.1007/s40415-020-00588-6

[B110] Shaw W. H. Anderson I. W. (1972). Purification, properties, and substrate specificities of ATP sulfurylase from spinach leaf tissue. *Biochem. J.* 127 237–247. 10.1042/bj1270237 5073745 PMC1178578

[B111] Shibuya K. Yamada T. Ichimura K. (2016). Morphological changes in senescing petal cells and the regulatory mechanism of petal senescence. *J. Exp. Bot.* 67 5909–5918. 10.1093/jxb/erw337 27625416

[B112] Silva N. C. Q. de Souza G. A. Pimenta T. M. Brito F. A. L. Picoli E. A. T. Zsögön A. (2018). Salt stress inhibits germination of Stylosanthes humilis seeds through abscisic acid accumulation and associated changes in ethylene production. *Plant Physiol. Biochem.* 130 399–407. 10.1016/j.plaphy.2018.07.025 30064096

[B113] Sisler E. C. Blankenship S. M. Fearn J. C. Haynes R. (1993). “Effect of diazocyclopen-tadiene (DACP) on cut carnations,” in *Cellular and Molecular Aspects of the Plant Hormone Ethylene*, Vol. 16 eds Pech J. C. Latche A. Balague C. (Dordrecht: Springer), 182–187.

[B114] Sisler E. C. Blankenship S. M. Guest M. (1990). Competition of cyclooctenes and cyclooctadienes for ethylene binding and activity in plants. *Plant Growth Regul.* 9 157–164. 10.1007/BF00027443

[B115] Sisler E. C. Serek M. (1997). Inhibitors of ethylene responses in plants at the receptor level: recent developments. *Physiol. Plant* 100 577–582. 10.1111/j.1399-3054.1997.tb03063.x

[B116] Sisler E. C. Serek M. Dupille E. Goren R. (1999). Inhibition of ethylene responses by 1-methylcyclopropene and 3-methylcyclopropene. *Plant Growth Regul.* 27 105–111. 10.1023/A:1006153016409

[B117] Sisler E. C. Yang S. F. (1984). Anti-ethylene effects of cis-2-butene and cyclic olefins. *Phytochemistry* 23 2765–2768. 10.1016/0031-9422(84)83011-3

[B118] Sors T. G. Ellis D. R. Na G. N. Lahner B. Lee S. Leustek T. (2005). Analysis of sulfur and selenium assimilation in *Astragalus* plants with varying capacities to accumulate selenium. *Plant J.* 42 785–797. 10.1111/j.1365-313x.2005.02413.x 15941393

[B119] Steffens B. (2014). The role of ethylene and ROS in salinity, heavy metal, and flooding responses in rice. *Front. Plant Sci.* 5:685 10.3389/fpls.2014.00685 PMC4255495 25538719

[B120] Su J. Nie Y. Zhao G. Cheng D. Wang R. Chen J. (2019). Endogenous hydrogen gas delays petal senescence and extends the vase life of lisianthus cut flowers. *Postharvest Biol. Tecnol.* 147 148–155. 10.1016/j.postharvbio.2018.09.018

[B121] Tagmount A. Berken A. Terry N. (2002). An essential role of S-adenosyl-L-methionine:L-methionine S-methyltransferase in selenium volatilization by plants. Methylation of selenomethionine to selenium-methyl-L-selenium methionine, the precursor of volatile selenium. *Plant Physiol.* 130 847–856. 10.1104/pp.001693 12376649 PMC166611

[B122] Terry N. Zayed A. M. de Souza M. P. Tarun A. S. (2000). Selenium in higher plants. *Annu. Rev. Plant Physiol. Plant Mol. Biol.* 51 401–432. 10.1146/annurev.arplant.51.1.401 15012198

[B123] Thao N. P. Khan M. I. R. Thu N. B. A. Hoang X. L. T. Asgher M. Khan N. A. (2015). Role of ethylene and its cross talk with other signaling molecules in plant responses to heavy metal stress. *Plant Physiol.* 169 73–84. 10.1104/pp.15.00663 26246451 PMC4577409

[B124] Tognon G. B. Sanmartin C. Alcolea V. Cuquel F. L. Goicoechea N. (2016). Mycorrhizal inoculation and/or selenium application affect post-harvest performance of snapdragon flowers. *Plant Growth Regul.* 78 389–400. 10.1007/s10725-015-0100-8

[B125] Ueda H. Kusaba M. (2015). Strigolactone regulates leaf senescence in concert with ethylene in *Arabidopsis*. *Plant Physiol.* 169 138–147. 10.1104/pp.15.00325 25979917 PMC4577378

[B126] Van Doorn W. G. (2001). Categories of petal senescence and abscission: a re-evaluation. *Ann. Bot.* 87 447–456. 10.1006/anbo.2000.1357

[B127] Van Doorn W. G. (2002). Effect of ethylene on flower abscission: a survey. *Ann. Bot.* 89 689–693. 10.1093/aob/mcf124 12102524 PMC4233834

[B128] Van Meeteren U. Aliniaeifard S. (2016). “Stomata and postharvest physiology,” in *Postharvest Ripening Physiology of Crops*, ed. Pareek S. (Boca Raton, FL: CRC Press), 157–216.

[B129] Veen H. (1979). Effects of silver on ethylene synthesis and action in cut carnations. *Planta* 145 467–470. 10.1007/BF00380101 24317863

[B130] Wallenberg M. Olm E. Hebert C. Björnstedt M. Fernandes A. P. (2010). Selenium compounds are substrates for glutaredoxins: a novel pathway for selenium metabolism and a potential mechanism for selenium-mediated cytotoxicity. *Biochem. J.* 429 85–93. 10.1042/BJ20100368 20408818

[B131] Wang C. Y. Baker J. E. Hardenburg R. Lieberman M. (1977). Effects of two analogs of rhizobitoxine sodium benzoate on senescence of snapdragons. *J. Am. Soc. Hortic. Sci.* 102 517–520.

[B132] Wang H. Woodson W. R. (1989). Reversible inhibition of ethylene action and interruption of petal senescence in carnation flowers by norbornadiene. *Plant Physiol.* 89 434–438. 10.1104/pp.89.2.434 16666561 PMC1055859

[B133] Wang K. L. C. Li H. Ecker J. R. (2002). Ethylene biosynthesis and signalling networks. *Plant Cell* 14 131–151. 10.1105/tpc.001768 PMC151252 12045274

[B134] Wang Y. Liu C. Li K. Sum F. Hu Z. Li X. (2007). Arabidopsis EIN2 modulates stress response through abscisic acid response pathway. *Plant Mol. Biol.* 64 633–644. 10.1007/s11103-007-9182-7 17533512

[B135] Wang Y. Zhao H. Liu C. Cui G. Qu L. Bao M. (2020). Integrating physiological and metabolites analysis to identify ethylene involvement in petal senescence in Tulipa gesneriana. *Plant Physiol. Biochem.* 149 121–131. 10.1016/j.plaphy.2020.02.001 32062332

[B136] White P. J. Bowen H. C. Parmaguru P. Fritz M. Spracklen W. P. Spiby R. E. (2004). Interactions between selenium and sulphur nutrition in *Arabidopsis thaliana*. *J. Exp. Bot.* 55 1927–1937. 10.1093/jxb/erh192 15258164

[B137] Wilson R. L. Kim H. Bakshi A. Binder B. M. (2014). The ethylene receptors ETHYLENE RESPONSE1 and ETHYLENE RESPONSE2 have contrasting roles in seed germination of *Arabidopsis* during salt stress. *Plant Physiol.* 165 1353–1366. 10.1104/pp.114.241695 24820022 PMC4081342

[B138] Wu F. Zhang C. Wang X. Guo J. Dong L. (2017). Ethylene-influenced development of tree peony cut flowers and characterization of genes involved in ethylene biosynthesis and perception. *Postharvest Biol. Tecnol.* 125 150–160. 10.1016/j.postharvbio.2016.11.014

[B139] Wuriyanghan H. Zhang B. Cao W.-H. Ma B. Lei G. Liu Y.-F. (2009). The ethylene receptor ETR2 delays floral transition and affects starch accumulation in rice. *Plant Cell* 21 1473–1494. 10.1105/tpc.108.065391 19417056 PMC2700534

[B140] Xue J. Huang Z. Wang S. Xue Y. Ren X. Zeng X. (2020). Dry storage improves the vase quality of cut peony by increasing water uptake efficiency through aquaporins regulation. *Plant Physiol. Biochem.* 148 63–69. 10.1016/j.plaphy.2020.01.007 31945668

[B141] Yamasaki S. Fujii N. Matsuura S. Mizusawa H. Takahashi H. (2001). The M locus and ethylene-controlled sex determination in andromonoecious cucumber plants. *Plant Cell Physiol.* 42 608–619. 10.1093/pcp/pce076 11427680

[B142] Yang S. Hoffman F. (1984). Ethylene biosynthesis and its regulation in higher plants. *Annu. Rev. Physiol.* 35 155–189. 10.1146/annurev.pp.35.060184.001103

[B143] Zeng Z. Jiang H. Zhang H. Jiang Z. (2012). The synthesis of novel oxime ethers and their effects on the senescence of cut carnation flowers. *Res. Chem. Intermed.* 38 463–470. 10.1007/s11164-011-0363-2

[B144] Zhang M. Smith J. A. C. Harberd N. P. Jiang C. (2016). The regulatory roles of ethylene and reactive oxygen species (ROS) in plant salt stress responses. *Plant Mol. Biol.* 91 651–659. 10.1007/s11103-016-0488-1 27233644

[B145] Zhu Z. Chen Y. Shi G. Zhang X. (2017). Selenium delays tomato fruit ripening by inhibiting ethylene biosynthesis and enhancing the antioxidant defense system. *Food Chem.* 219 179–184. 10.1016/j.foodchem.2016.09.138 27765214

